# Performance Assessment of Change Detection Based on Robust PCA for Wavelength Resolution SAR Images Using Nonidentical Flight Passes

**DOI:** 10.3390/s25082506

**Published:** 2025-04-16

**Authors:** Lucas P. Ramos, Viet T. Vu, Mats I. Pettersson, Patrik Dammert, Leonardo T. Duarte, Renato Machado

**Affiliations:** 1 Department of Telecommunications, Aeronautics Institute of Technology, São José dos Campos 12228-900, Brazil; rmachado@ita.br; 2 Department of Mathematics and Natural Sciences, Blekinge Institute of Technology, 371 79 Karlskrona, Sweden; viet.thuy.vu@bth.se (V.T.V.); mats.pettersson@bth.se (M.I.P.); 3 Saab Surveillance, Saab AB, 412 89 Gothenburg, Sweden; patrik.dammert@saabgroup.com; 4 School of Applied Sciences, State University of Campinas, Limeira 13484-350, Brazil; leonardo.duarte@fca.unicamp.br

**Keywords:** change detection, nonidentical passes, RPCA, SAR, wavelength resolution

## Abstract

One of the main challenges in Synthetic Aperture Radar (SAR) change detection involves using SAR images from different flight passes. Depending on the flight pass, objects have different specular reflections since the radar cross-sections of these objects can be totally different between passes. Then, it is common knowledge that the flight passes must be close to identical for conventional SAR change detection. Wavelength-resolution SAR refers to a SAR system with a spatial resolution approximately equal to the wavelength. This high relative resolution helps to stabilize the ground clutter in the SAR images. Consequently, the restricted requirement about identical flight passes for SAR change detection can be relaxed, and SAR change detection becomes possible with nonidentical passes. This paper shows that robust principal component analysis (RPCA) is efficient for change detection even using wavelength-resolution SAR images acquired with very different flight passes. It presents several SAR change detection experimental results using flight pass differences up to 95°. For slightly different passes, e.g., 5°, our method reached a false alarm rate (FAR) of approximately one false alarm per square kilometer for a probability of detection (PD) above 90%. In a particular setting, it achieves a PD of 97.5% for a FAR of 0.917 false alarms per square kilometer, even using SAR images acquired with nonidentical passes.

## 1. Introduction

Due to the system’s characteristics of monitoring the Earth independently of sunlight conditions and cloud coverage, Synthetic Aperture Radar (SAR) systems have become an ideal source for change detection applications [1]. SAR change detection enables the identification of changes from multi-temporal images acquired in the same scene. This change may represent a target that emerged or disappeared in the scene over time. This application is relevant in remote sensing, allowing, for example, monitoring environmental changes and land use/cover [2].

Change detection methods based on wavelength-resolution SAR systems have been explored for the past two decades [3,4,5]. Since the system resolution is relative to the radar signal wavelength, the main contribution to SAR image clutter (i.e., return relating to the acquisition environment), especially at low frequencies, arises from large static objects (i.e., large scatterers), which are stable over time. Thus, the measurements carried out by this system become stable in different acquisitions [6]. This is desirable for change detection applications since it allows for obtaining images of a given area with similar characteristics, i.e., highly correlated.

Traditionally, one of the main challenges for SAR change detection involves exploring the use of SAR images acquired from nonidentical flight passes, meaning that the SAR aperture is formed based on different flight headings. For example, the radar cross-section can be totally different, as it depends on how the target reflects the radar signal, which depends on the flight pass. In addition, power lines, fences, and buildings contribute with a very strong specular reflection depending on the pass, increasing the number of false alarms [7]. Recently, some studies have sought to use different passes in change detection, mainly exploring the stability of wavelength-resolution systems. In [7,8], the nonidentical passes of the SAR measurements are suggested to be combined to enhance SAR change detection performance. This combination allows for mitigating specular reflections, mainly from elongated structures in the SAR scene, and interference due to the antenna back lobe. Thus, the probability of detection has been shown to improve, whereas the false alarm rate is minimized.

In fact, the SAR change methods found in the literature usually use co-registration steps to avoid misalignments between the SAR images [9,10,11]. In low-frequency SAR, like wavelength-resolution SAR, some studies combined identical flight passes to explore different flight passes [7,8]. Such methods explore statistical hypothesis testing in which a decision threshold must be applied, which will decide whether a pixel is related to the target or clutter. It is very difficult for an approach like this to differentiate whether detection is associated with a true target or some reflection related to using nonidentical flight passes.

This paper shows that the identical pass constraint can be relaxed for wavelength-resolution SAR change detection based on the robust principal component analysis (RPCA) [12]. RPCA is a blind signal separation technique recently introduced for change detection [13], in which the input data are decomposed into low-rank and sparse components. Generally, since the target tends to move over time, the sparse component will detect this object and other sparse objects, such as power lines, related to nonidentical flight passes. In other words, RPCA detects both targets and reflections related to flight passes, which can be disregarded from the false alarm count because they usually present a characteristic behavior in the SAR image (elongated structures), thus improving the method’s performance. In addition, using more reference SAR images with such reflections related to the flight passes will make this content appear in the low-rank component, i.e., becoming something related to the clutter, which would be the ideal scenario since those structures are not the targets of interest for the application.

It is important to note that this study does not aim to propose a new change detection method based on RPCA. Instead, it aims to show another potential in low-frequency SAR that has not been demonstrated before: exploring different flight passes in a wavelength-resolution SAR change detection method based on RPCA. This study is based on the fact that the RPCA is a matrix solution that looks at the data matrix as a whole and can then detect changes related to targets and associated with using different flight passes. At the same time, reference wavelength-resolution SAR change detection methods are usually based on testing statistics, such as amplitude ratio and generalized likelihood ratio tests [4,7,8], which are based on a pair of images using the one-look data statistics and are not able to perform change detection on nonidentical flight pass since the statistics is totally different when the pass changes.

We evaluate the surveillance and reference images in three scenarios: SAR images obtained from identical, slightly different, and totally different passes. Slightly different means a difference of 5° between the pass of the surveillance image and reference images, while totally different means a difference of 90° or 95°. We consider the dataset formed by 24 coregistered SAR images from the wavelength-resolution CARABAS II SAR system [3].

This article is organized as follows. Section 2 presents the classical formulation of RPCA and the recently proposed change detection methods based on RPCA. Section 2.3 presents the CARABAS data with identical and nonidentical passes. Then, Section 3 shows and evaluates detection results in terms of probability of detection and false alarm rate, considering different arrangements for the data. A discussion about the results obtained is presented in Section 4. Section 5 summarizes the conclusions of the paper.

## 2. Materials and Methods

This section presents the RPCA formulation and recent change detection methods in SAR images based on RPCA. Then, we show how the data are organized for a change detection method based on a matrix approach such as RPCA. Finally, the dataset used in this study is presented.

### 2.1. PCA and RPCA

Principal component analysis (PCA) is usually used to handle tasks such as linear dimensionality reduction, image denoising, and image clustering [14]. PCA transforms the original data into new features known as principal components (PCs). The PCs represent linear combinations of the initial variables, effectively capturing the maximum variance across all variables [15]. Then, it is possible to approximate the original dataset by utilizing only these PCs, which are sensitive to noise, outliers, and missing data in the original data [16].

Different approaches in the literature address the limitation of the PCA [15]. Most of them are based on decomposing the input data X into a low-rank L and a sparse matrix S, which is characterized by a small fraction of non-zero entries such as outliers and missing data [17]. In this context, RPCA via principal component pursuit (PCP) [12] has become very popular [18]. Under certain assumptions [12], it is possible to recover L even with arbitrarily large magnitude in S, based on the following convex optimization problem given by

(1) $$\begin{array}{cc} \mathrm{minimize} & {∥L∥}_{*}+\lambda {∥S∥}_{1}\\ \;\mathrm{subject}\;\mathrm{to}\; & L+S=X \end{array},$$

where ${∥L∥}_{*}$ is the nuclear norm of L, i.e., sum of all singular values. This norm is the tightest convex relaxation for low-rank matrix functions [19]. ${∥S∥}_{1}$ is the $l_{1}$-norm of S and represents the sum of the absolute values of all entries. In this formulation, λ controls the amount of information contained in each matrix and is usually defined by $\lambda =1/\sqrt{\max(n,m)}$, where *n* and *m* are the size of X.

Convex optimization problems, such as those found in the RPCA (1), can be solved with a theoretical convergence guarantee in different ways [20]. In our analysis, we use the Alternating Direction Method of Multipliers (ADMM) method [20]. The ADMM method’s main advantage is exploring the additive characteristic of the decomposition and alternatively performing the optimization problem. Thus, the problem can be split into subproblems that are generally easier to compute [18,20]. The RPCA problem solved by the ADMM method can be written as

(2) $$\left\{\begin{array}{cc} L^{\left(t+1\right)} & =\underset{L}{\arg\min}\left\{{∥L∥}_{*}\right.\\ \; & \left.+\frac{\mu^{\left(t\right)}}{2}{∥L+S^{\left(t\right)}-X+\frac{\Lambda^{\left(t\right)}}{\mu^{\left(t\right)}}∥}_{F}^{2}\right\},\\ S^{\left(t+1\right)} & =\underset{S}{\arg\min}\left\{{∥S∥}_{1}\right.\\ \; & \left.+\frac{\mu^{\left(t\right)}}{2}{∥L^{\left(t+1\right)}+S-X+\frac{\Lambda^{\left(t\right)}}{\mu^{\left(t\right)}}∥}_{F}^{2}\right\},\\ \Lambda^{\left(t+1\right)} & =\Lambda^{\left(t\right)}+\mu^{\left(t\right)}\left(L^{\left(t+1\right)}+S^{\left(t+1\right)}-X\right),\\ \mu^{\left(t+1\right)} & =\min(\rho \mu^{\left(t\right)},\mu_{max}), \end{array}\right.$$

where Λ represents the Lagrange multiplier matrix [21], μ>0 denotes the called penalty parameter [22], and ρ is a scalar used to increase the penalty μ up to $\mu_{max}$ and, consequently, reduce the convergence time of the method [23].

### 2.2. SAR Change Detection Based on RPCA

Wavelength-resolution SAR images are generally known not to suffer from fully developed speckle noise since a single scatter usually forms the resolution cell [6]. Therefore, it can be assumed that the composition of wavelength-resolution SAR images occurs in a superposition process in the complex domain of signals radiated by different sources such as targets, clutter, and noise. Consequently, unsupervised separation techniques can be employed. Recently, [13] introduced RPCA via PCP in the context of wavelength-resolution SAR change detection using SAR image pairs from identical passes acquired at different moments. The motivation for the study lies in the sparse nature of the targets, represented by the sparse content of the decomposition, i.e., S. The approach in [13] is considered a baseline for SAR change detection based on RPCA and shows that targets can be detected in a surveillance image, and false alarms can be reduced by analyzing the sparse content of the reference image.

Motivated by using SAR image stacks in change detection methods, [24] extended [13] on small stacks of wavelength-resolution SAR images. The authors formed the stack with images from identical and nonidentical passes to explore temporal diversity. This approach naturally increases the number of false alarms due to structures sensitive to the flight pass. Therefore, a heuristic based on three rules was developed to remove false alarms and make the change detection method efficient, involving a configurable parameter that allowed a high-performance gain.

As a matrix solution, both RPCA-based change detection methods concatenate the SAR images to explore the diversity present in the stack. According to [13], there are no gains in terms of detection when concatenating the rows or columns of the input SAR images. In our study, we concatenated the rows of the SAR images, creating a matrix X with *n* SAR images given by

(3) $$X=\left[\begin{array}{c} X_{1}\\ X_{2}\\ \vdots \\ X_{n} \end{array}\right]=\left[\begin{array}{cccc} p_{11} & p_{12} & \ldots & p_{1m}\\ p_{21} & p_{22} & \ldots & p_{2m}\\ \vdots & \vdots & \ddots & \vdots \\ p_{n1} & p_{n2} & \ldots & p_{nm} \end{array}\right],$$

where m=1,2,⋯,r×c are the pixels of the SAR image with *r*-rows and *c*-columns representing the dimension of each SAR image. Since the number of pixels usually is much greater than the number of images, i.e., m≫n, the λ value defined in the formulation of the RPCA [12] can be rewritten as $\lambda =1/\sqrt{\max(n,m)}\;=\;1/\sqrt{rc}$. It is important to note that the RPCA formulation depends on the stack being formed by SAR images of the same dimension, so the reference SAR images must be of the same dimension as the surveillance SAR image.

### 2.3. CARABAS Data with Identical and Nonidentical Passes

CARABAS is a Swedish SAR system operating in the 20–90 MHz band. Due to the frequency of operation, the main contribution to the radar reflections comes from man-made objects, such as vehicles and buildings, according to the wavelength. This characteristic makes this system well suited for civil and military applications, as it is possible to detect these objects even though they are concealed by forest and under camouflage [8].

In 2002, a measurement campaign was carried out using the CARABAS II system in a region called RFN Vidsel, Sweden. In that case, 25 military vehicles (i.e., targets) hidden under foliage were deployed in two different forests in the northwest and southeast of the imaged scene. The data obtained from this measurement were made available in [25], and since then, many studies have been working with such data to mainly propose SAR change detection [3,4,6,7,8]. The data are formed by 24 SAR images with 3000 rows and 2000 columns each. These images cover the same geographic area of 6 km^2^ (3 km in azimuth × 2 km in range), and the spatial resolution is around 2.5 m in both dimensions.

The dataset is divided by SAR images acquired with three different flight passes, i.e., eight SAR images from each flight pass. In a traditional microwave SAR system, these different flight passes will result in very different scattering statistics on the ground. Most scattering on targets occurs at trihedral reflections, and these reflections will appear and disappear for other passes. Clutter is also very sensitive due to speckle de-correlating in terms of time and flight path. However, this differs for wavelength-resolution systems operating at low frequencies with no speckle in the images and non-trihedral reflection on targets. Unfortunately, high bandwidth at Very High Frequency (VHF) is associated with radio frequency interference (RFI).

The flight passes in this study represent 135°, 225°, and 230°. The flight passes present different intensities of RFI, so those where the main lobe of the radar antenna points towards a nearby television station, i.e., passes of 225° and 230°, are those most affected by RFI [3]. In addition, each of the three flight passes has four different target deployments, with two SAR images for each deployment. The deployments are called missions 2, 3, 4, and 5. In missions 2 and 3, the targets are placed in a forest in the northwest region of the scene, while for missions 4 and 5, the targets are placed in a forest in the southeast region.

**Figure 1:**
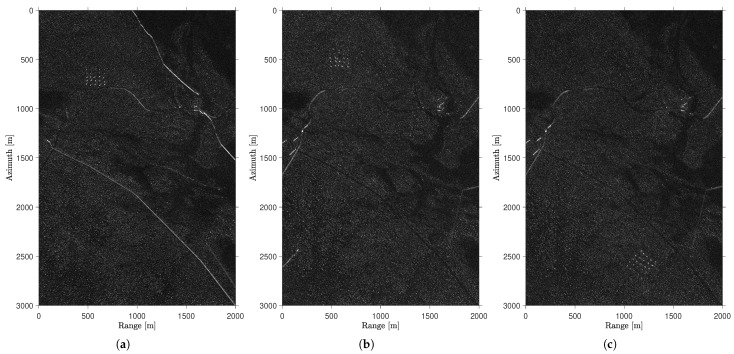
CARABAS-II data with different target deployments and different passes. (**a**) mission 2 acquired with 135°, (**b**) mission 3 acquired with 225°, and (**c**) mission 5 acquired with 230°.

**Figure 2:**
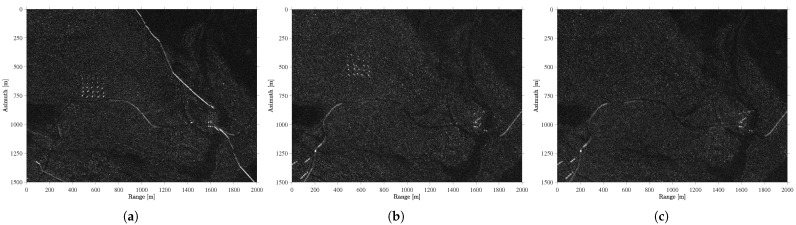
Figure 1 in the north up region [0–1500] in Azimuth. (**a**) mission 2 acquired with 135°, (**b**) mission 3 acquired with 225°, and (**c**) mission 5 acquired with 230°.

Figure 1 shows the magnitude of the ground scene from the CARABAS data, considering three different passes. Initially, we can observe that the targets are deployed in different positions (i.e., deployments). We can also highlight the importance of the pass in acquiring SAR images. In Figure 1a, in which a pass of 135° is considered, reflections related to elongated structures can be observed in the range direction, between 1000 and 2000 m. However, when performing measurements with a pass of 230°, as shown in Figure 1c, some reflections are present between 0 and 500 m in the range direction. Since these elongated structures remain stable over time, detecting targets is possible when using SAR images from identical passes. On the other hand, detecting targets becomes challenging when working with SAR images obtained from different passes, as such structures significantly increase the number of detections related to false alarms. Those elongated structures can be clearly seen in Figure 2 which shows the north up region of Figure 1.

Since the 24 SAR images from the CARABAS dataset [3] are formed by eight SAR images from three different flights (135°, 225°, 230°), we can arrange the images as follows for the experiments: identical passes, slightly different passes, and totally different passes. Identical pass means considering surveillance and reference images from the same pass in the change detection method. In this case, we have one surveillance image and seven reference images from the same pass (i.e., *n* = 8). Slightly different passes consider surveillance and reference SAR images from 225° and 230°, and totally different is when surveillance and reference images come from 135° and 225° or 135° and 230°. In the slightly and totally different analysis, *n* = 9. This is justified because, for these analyses, we have a surveillance image from a given pass and eight reference images from either slightly or totally different passes, depending on the case. Table 1 summarizes the arrangements considered to evaluate our analysis.

## 3. Experimental Results

**Figure 3:**
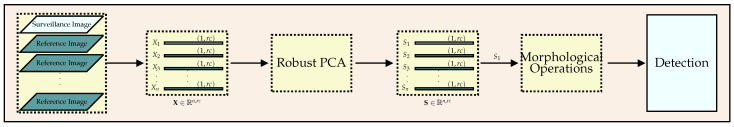
Change detection based on RPCA.

Here, we present an experiment to perform change detection on CARABAS data based on RPCA. The framework of the experiment is presented in Figure 3. The change detection approach is based on identifying targets in a surveillance image by analyzing the respective sparse content, denoted as $S_{1}$. For that, we consider reference SAR images from identical, slightly different, and totally different passes.

Each surveillance SAR image is evaluated based on the probability of detection (PD) and false alarm rate (FAR) [7,8]. PD is obtained from the ratio between the number of detected targets and the known 25 military vehicles on the scene. Any detection not related to the targets is considered a false alarm (FA), and then the FAR is calculated by the ratio between FA and the area of the scene, i.e., $6\;{\mathrm{km}}^{2}$.

PD and FAR values are obtained by considering different amounts of information in $S_{1}$. Hence, it is important to range the λ value. In [13,24], the results show that the best performance in terms of PD and FAR on CARABAS data is obtained when λ varies between 7 and 10 times the reference λ value [12]. The λ value on the CARABAS data is given by $1/\sqrt{rc}=1/\sqrt{3000\cdot 2000}=0.408\cdot 10^{-3}$. In our study, we range this value from 1 up to 13 times with a step size of 1. The work in [26] is considered as the reference for the implementation of the RPCA, and then the following parameters are used: $\mu =10\lambda ,\;\mu_{max}=10^{10}$, and ρ=1.1. Those values are related to the ADMM optimization method that solves RPCA and will mainly define the convergence rate of the technique. More details on how to define such values can be seen in [22,23].

Similar to other wavelength-resolution change detection methods [3], we consider morphological operations of opening and dilation based on the spatial resolution of the CARABAS data. From that, removing objects smaller than the system’s spatial resolution and connecting local changes separated by less than the practical spatial resolution is possible.

### 3.1. Identical Passes

Initially, we evaluate change detection performance using images from the same pass (n=8). Table 2 shows the results obtained in terms of PD and FAR for $5/\sqrt{rc}$. Note that when a SAR image is used for surveillance, the remaining seven images are considered reference images in the stack. In this particular setting, we obtain a PD of 96.5% and FAR = 0.250 $\mathrm{FA}/{\mathrm{km}}^{2}$ when considering surveillance images from 225°. Considering the average of the three flight tracks, the method reaches a PD of 95.33% for a FAR of 0.451 $\mathrm{FA}/{\mathrm{km}}^{2}$. The result obtained is superior to the reference method proposed in the challenge problem [3] and competitive to the ones presented in [24]. However, the method in [24] is considered a rule to eliminate false alarms, which is not used in this study. Finally, we can observe that a single surveillance image with a low detection probability can significantly influence performance. For example, Image 4 of the stack with surveillance images from 230° detects only 15 targets and 7 false alarms, which accounts for almost 50% of the total 15 false alarms.

**Figure 4:**
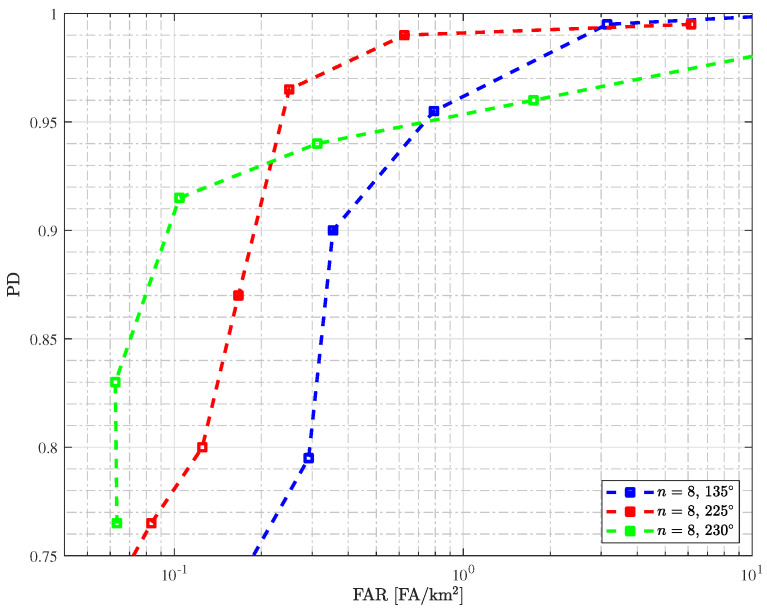
ROC performance of the change detection using surveillance and reference SAR images from identical passes (n=8).

Figure 4 shows receiver operating characteristic (ROC) curves in terms of PD and FAR. The curves are obtained by varying the λ value, and each PD and FAR value in the ROC curve represents an average value of the eight surveillance images as presented in Table 2. For a PD of about 90%, it is possible to obtain a FAR = 0.104 $\mathrm{FA}/{\mathrm{km}}^{2}$ when using images from 230° and FAR = 0.354 $\mathrm{FA}/{\mathrm{km}}^{2}$ when using images from 135°. Finally, in all cases using identical passes, obtaining a PD of at least 95% for a FAR of 1 $\mathrm{FA}/{\mathrm{km}}^{2}$ is excellent performance for change detection.

### 3.2. Slightly Different Passes

**Figure 5:**
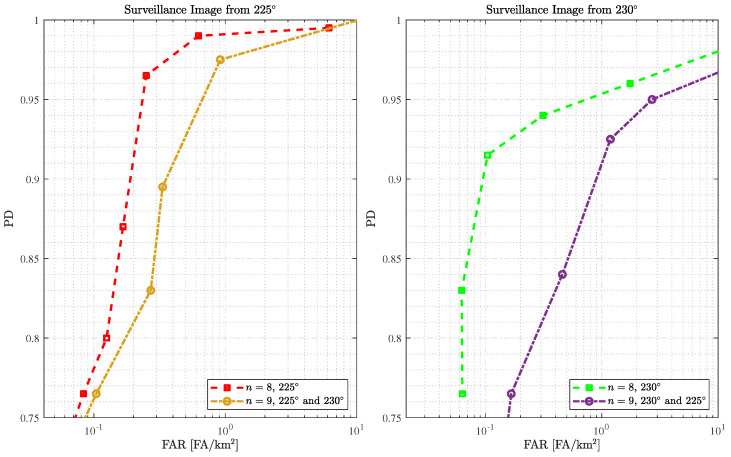
ROC performance of the change detection using surveillance and reference SAR images from identical and slightly different passes, i.e., n=8 and n=9, respectively.

The second analysis uses reference images from slightly different passes (n=9). Figure 5 shows the results obtained using surveillance images from 225° and 230° through ROC curves and compares them with those obtained with identical passes. Note that we are evaluating the same eight surveillance images as in the analysis of identical passes, changing only the reference images: seven reference images from the same pass and eight reference images from slightly different passes for identical and slightly different passes, respectively.

As expected, the best performance is obtained using identical passes. However, we achieve outstanding performance even when using slightly different passes. For example, a PD of 97.5% and FAR = 0.917 $\mathrm{FA}/{\mathrm{km}}^{2}$ is obtained when using surveillance images from 225° and reference images from 230°. In addition, the results show that 1.187 $\mathrm{FA}/{\mathrm{km}}^{2}$ is obtained with 92.5% of PD when using surveillance images from 230° and reference images from 225°. This study demonstrates that small variations in flight passes can still be accepted for change detection, reinforcing the stability of wavelength-resolution SAR systems and their efficacy.

If we compare the results using the surveillance image from 225° and reference images from 230° (i.e., yellow curve) to results using SAR images from an identical pass from the 230° (i.e., green curve), we can observe that for a FAR of approximately 1 $\mathrm{FA}/{\mathrm{km}}^{2}$, a better PD is reached using the approach based on slightly different passes. These results show that better performance can still be obtained considering the slightly different passes, depending on the surveillance SAR images considered.

### 3.3. Totally Different Passes

**Figure 6:**
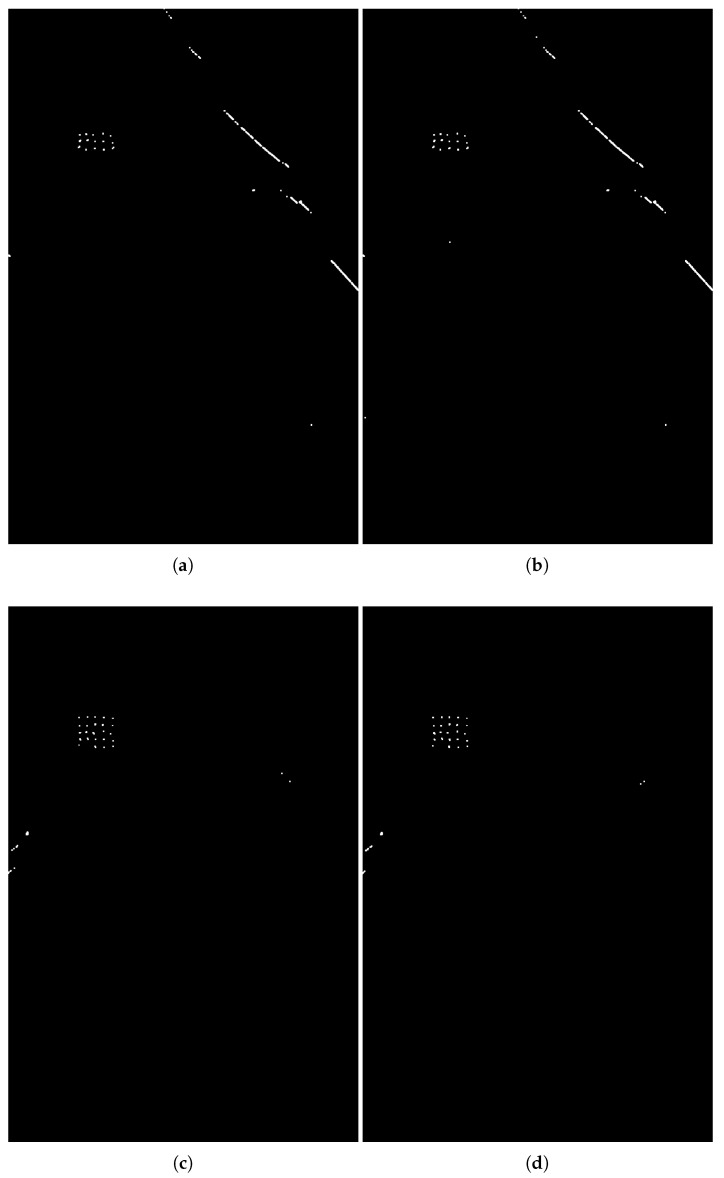
Change detection results on CARABAS-II data using totally different passes (n=9) for $\lambda =2.8577\cdot 10^{-3}$. (**a**) surveillance image from mission 2 acquired with 135° and eight reference images from 225°, (**b**) surveillance image from mission 2 acquired with 135° and eight reference images from 230°, (**c**) surveillance image from mission 2 acquired with 225° and eight reference images from 135°, and (**d**) surveillance image from mission 2 acquired with 230° and eight reference images from 135°.

This section presents the change detection performance in scenarios where reference images are acquired with totally different passes (n=9). The flight pass is changed up to 95°. Figure 6 shows the detection results considering the four possible arrangements of totally different passes using CARABAS data for $\lambda =7/\sqrt{rc}$ (i.e., $2.8577\cdot 10^{-3}$). Targets can be detected very well due to the high degree of correlation between the wavelength-resolution SAR data, reaching a PD of 96% in specific cases (Figure 6c,d). Furthermore, a reduction in λ can allow better performance in terms of PD.

However, there is a significant increase in the occurrence of false alarms, mainly related to elongated structures that are sensitive to the flight pass. These elongated structures are presented in Figure 6 and are detected due to their sparse nature in the input matrix. Using reference images acquired with a closer flight pass to the surveillance image (e.g., identical and slightly different passes) will ensure that these structures are not sparse and, therefore, will not be represented in the sparse content of the surveillance image.

**Figure 7:**
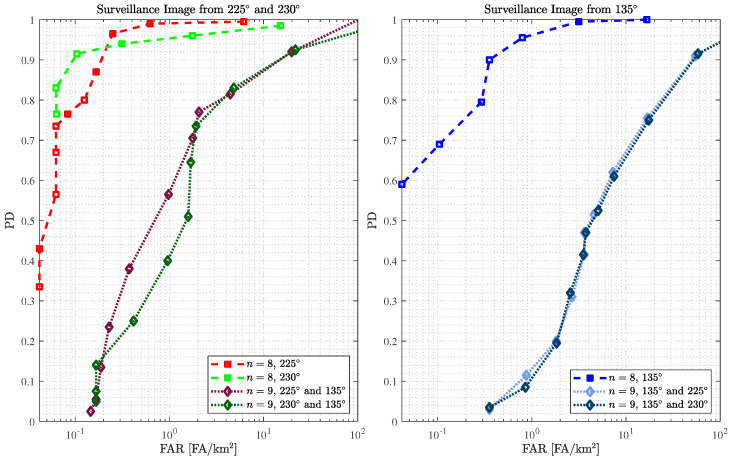
ROC performance of the change detection using surveillance and reference SAR images from identical and totally different passes, i.e., n=8 and n=9, respectively. Most false alarms are related to elongated man-made objects that can be mitigated by alternative methods.

Figure 7 presents change detection performance in terms of ROC curves using reference images from totally different passes and compares them with those with identical passes. The results show that change detection is feasible even using totally different passes in wavelength-resolution SAR systems. For cases with surveillance images of 225° and 230°, a degradation in PD of approximately 23% is observed compared to change detection using identical passes. For example, for a FAR of approximately 2 $\mathrm{FA}/{\mathrm{km}}^{2}$, there is a decrease in the PD from 99.0% to 77.0% and from 96.0% to 73.5% for surveillance images of 225° and 230°, respectively. An even more significant degradation of approximately 53% is found when surveillance images from 135° are used. However, remember that false alarms are mainly related to elongated man-made structures that present a pattern in the sparse SAR image (see mainly Figure 1a). It could easily be classified by the system operator as content that is not, in fact, a target and then disregarded from the analysis. In this way, the effectiveness of wavelength-resolution SAR systems in change detection is shown even in worst-case scenarios, i.e., when the surveillance image is acquired with a pass totally different from the reference.

## 4. Discussion

Our study mainly shows that blind signal separation techniques, in this case RPCA, are robust enough to allow the use of SAR images from different flight passes in a change detection method. In traditional change detection methods, the use of SAR images obtained with identical flight passes is a requirement. Such methods are not robust enough to work with SAR images from different flight passes. In addition, it is very difficult to guarantee exactly identical flight paths between measurements.

To evaluate the performance of the change detection method based on RPCA, we consider three different scenarios, one with reference images identical to the surveillance image, another one with reference images slightly different from the surveillance image, and the last one where the surveillance image is obtained with a flight pass totally different from the flight pass of the reference images. Comparing the scenarios, the results show that the use of identical images guarantees better performance in terms of PD and FAR. For the case where the reference images are slightly different, it is still possible to obtain a FAR lower than one false alarm per square kilometer for a PD greater than 90%. When working with a totally different flight pass of around 90°, there is a loss in performance. As shown in the figures, this loss is not associated with false alarms from forest backscatter but rather with false alarms related to elongated structures sensitive to flight pass.

The performance assessment of this paper is strictly based on the characteristics of the low-frequency SAR system (e.g., stability between measurements and SAR images non-affected by speckle noise and stripmap mode). Different systems operating at other frequencies such as TerraSAR-X and Sentinel-1 certainly face challenges that prevent using different flight passes in the change detection method. In future work, we aim to explore the challenges from other sensors to verify the method generalizability. In addition, RPCA can be used jointly with other post-detection approaches that allow differentiating false alarms related to forest backscatter from false alarms related to changes in flight pass. Finally, other blind signal separation techniques can be applied, aiming at better performance.

## 5. Conclusions

The restricted requirement about identical passes for SAR change detection can be relaxed for change detection with wavelength-resolution SAR. The experiments on CARABAS data show that change detection with nonidentical passes is feasible with wavelength-resolution SAR. For slightly different passes, e.g., with the flight pass of 225° and 230°, the detection probabilities are above 90% with a false alarm rate of 1 false alarm per square kilometer. Such change detection performance can be considered to be very good. Compared with the change detection performance using identical passes, the degradation in detection probability is less than 5%. In the case where the totally different passes are considered for change detection, the degradation in PD is about 23% for surveillance images with flight passes of 225° and 230° and 53% for surveillance images with flight passes of 135°. This degradation is mainly related to elongated structures sensitive to the flight path, such as transmission lines and fences. Using reference images acquired with a closer flight pass to the surveillance image (e.g., identical and slightly different passes) will ensure that these structures are not sparse and, therefore, will not be represented in the sparse content of the surveillance image. Despite a decrease in PD and an increase in false alarms, our studies show that it is possible to perform change detection using images from different flight passes, given that the forest backscatter in wavelength-resolution SAR is similar even with a 95° change in the flight pass.

## Figures and Tables

**Table 1:** CARABAS data arrangements.

Pass of Reference Images	Pass of Surveillance Image		
	135°	225°	230°
135°	Identical	Totally	Totally
225°	Totally	Identical	Slightly
230°	Totally	Slightly	Identical

**Table 2:** Change detection results in terms of PD and FAR based on identical passes for $5/\sqrt{rc}$.

Surveillance SAR Image	About the Scene		Flight Pass: 135°				Flight Pass: 225°				Flight Pass: 230°			
	Known Targets	Area [${\mathrm{km}}^{2}$]	Detected Targets	PD	FA	FAR [$\mathrm{FA}/{\mathrm{km}}^{2}$]	Detected Targets	PD	FA	FAR [$\mathrm{FA}/{\mathrm{km}}^{2}$]	Detected Targets	PD	FA	FAR [$\mathrm{FA}/{\mathrm{km}}^{2}$]
Mission 2, Image 1	25	6	25	1.000	2	0.333	25	1.000	0	0.000	25	1.000	0	0.000
Mission 2, Image 2	25	6	24	0.960	3	0.500	25	1.000	3	0.500	25	1.000	3	0.500
Mission 3, Image 3	25	6	25	1.000	4	0.667	25	1.000	3	0.500	15	0.600	7	1.167
Mission 3, Image 4	25	6	25	1.000	3	0.500	22	0.880	0	0.000	24	0.960	2	0.333
Mission 4, Image 5	25	6	25	1.000	8	1.333	25	1.000	0	0.000	25	1.000	1	0.167
Mission 4, Image 6	25	6	25	1.000	7	1.167	25	1.000	2	0.333	25	1.000	1	0.167
Mission 5, Image 7	25	6	22	0.880	7	1.167	23	0.920	4	0.667	24	0.960	0	0.000
Mission 5, Image 8	25	6	20	0.800	4	0.667	23	0.920	0	0.000	25	1.000	1	0.167
Total	200	48	191	0.955	38	0.792	193	0.965	12	0.250	188	0.940	15	0.3125

## Data Availability

The data that support the findings of this study are available from the Sensor Data Management System. Available online: https://www.sdms.afrl.af.mil (accessed on 3 May 2023).

## Abbreviations

The following abbreviations are used in this manuscript:

ADMM	Alternating Direction Method of Multipliers
FA	False Alarm
FAR	False Alarm Rate
PC	Principal Component
PCA	Principal Component Analysis
PCP	Principal Component Pursuit
PD	Probability of Detection
RFI	Radio Frequency Interference
ROC	Receiver Operating Characteristic
RPCA	Robust Principal Component Analysis
SAR	Synthetic Aperture Radar
VHF	Very High Frequency
